# A highly sensitive fluorimetric protocol based on isoindole formation for determination of gabapentin

**DOI:** 10.1039/c9ra06164a

**Published:** 2019-09-23

**Authors:** Tamer Z. Attia, Mohamed Elnady, Sayed M. Derayea

**Affiliations:** Department of Analytical Chemistry, Faculty of Pharmacy, Minia University Minia Egypt sayed_derayea@mu.edu.eg

## Abstract

A new, simple, highly sensitive and selective spectrofluorimetric method was developed for determination of gabapentin through its derivatization with *o*-phthalaldehyde in the presence of 2-mercaptoethanol. The resulting product was highly fluorescent and its emission intensity was measured at 431 nm after excitation at 335 nm. The effect of different experimental parameters on the formation and stability of the fluorescent product was carefully studied and optimized. The fluorescence–concentration plot was rectilinear over the range of 25–125 ng mL^−1^. The lower detection and quantification limits were 3.4 mL^−1^ and 11.2 ng mL^−1^, respectively. The procedure was fully validated according to the guidelines of the International Conference on Harmonization. The proposed method was successfully applied for the determination of the investigated drug in its pharmaceutical capsules and the results were in agreement with those of the reported method, in terms of the accuracy and precision. The low cost of analysis and high sensitivity make the proposed method ideally suited for analysis of the investigated drug in quality control laboratories.

## Abstract 2

A new, simple, highly sensitive and selective spectrofluorimetric method was developed for determination of gabapentin through its derivatization with *o*-phthalaldehyde in the presence of 2-mercaptoethanol.

## Graphical abstract

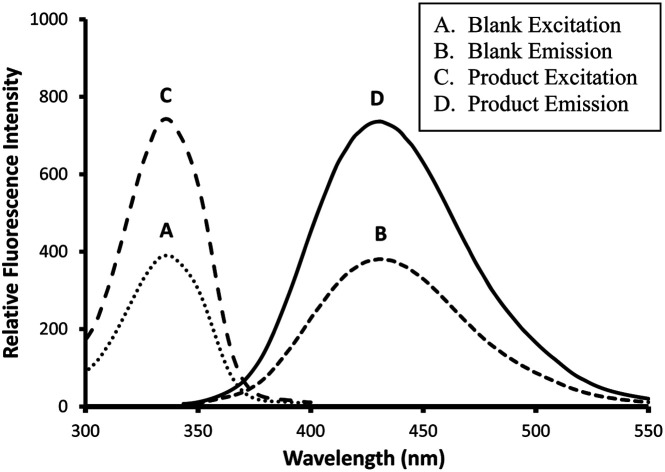

## 1. Introduction

**Fig. 1:**
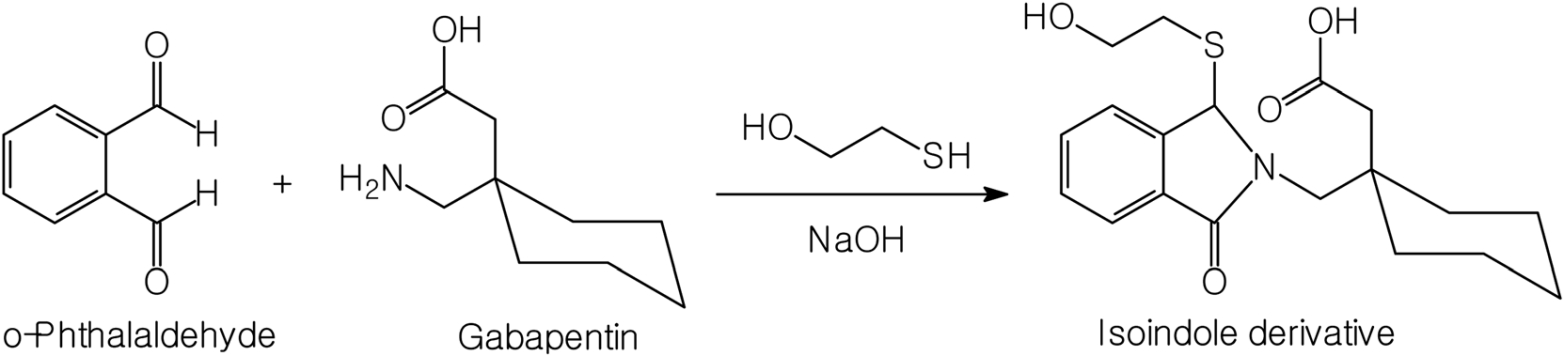
The proposed pathway of the reaction between gabapentin and *o*-phthalaldehyde/2-mercaptoethanol reagent.

Gabapentin (Fig. 1), chemically known as 1-(amino methyl)cyclohexane acetic acid, is a structural analogue of the inhibitory neurotransmitter gamma-amino butyric acid (GABA) and its action is attributed to the irreversible inhibition of the enzyme GABA-transaminase, thus preventing the physiological degradation of GABA in the brain. It is an anticonvulsant drug used in the treatment of epilepsy and neuropathic pain, as an adjunct therapy for partial seizures in adults and children.^1^

An analytical literature survey revealed that several methods have been reported for determination of gabapentin in pure forms and pharmaceutical dosage forms including spectrophotometry,^2–4^ spectrofluorimetry,^5–8^ high-performance liquid chromatography (HPLC),^9–11^ gas chromatography-mass spectrometry (GC-MS),^12^ capillary electrophoresis^13^ and electrochemical techniques.^14^

The reported spectrofluorimetric methods of analysis are either suffer from low sensitivity^5–7^ or require heating and tedious extraction procedures.^8^ In addition, the chromatographic, electrophoretic and electrochemical methods require highly sophisticated instruments and/or require expensive detector which is not available in most laboratories.

The aim of the present work is to develop a simple and highly sensitive spectrofluorimetric method for determination of gabapentin. The amino group of the investigated drug was condensed with *o*-phthalaldehyde to form a highly fluorescent product. The proposed method was applied for the determination of gabapentin in its capsules without any interference from the excipients. The advantage of spectrofluorimetric method over other methods of analysis is that it does not require highly sophisticated instruments and more sensitive than the reported spectrophotometric methods of analysis.

## 2. Experimental

### 2.1 Apparatus

PerkinElmer UK model LS 45 Luminescence Spectrometer was used for performing all the spectrofluorimetric measurements. The instrument is equipped with a 150 W xenon arc lamp and a 1 cm quartz cell, connected to an IBM PC computer loaded with the FL WINLAB™ software. Grating excitation and emission monochromators slit width for both monochromators were set at 10 nm.

Spectronic™ Genesys™ 2PC Ultraviolet/Visible Spectrophotometer (Milton Roy Co, USA) with a matched 1 cm quartz cell, connected to IBM computer loaded with Winspec™ application software. Milwaukee SM 101 pH meter, Portugal and Digital Analytical Balance (AG 29, Mettler Toledo, Glattbrugg, Switzerland) were also used.

### 2.2 Chemicals and reagents

Gabapentin was kindly provided by Delta Pharm Company (10^th^ of Ramadan city, El Sharkeya, Egypt). It was used without further purification. *o*-Phthalaldehyde (Loba Chemie, Pvt. Ltd. 107, Woodhouse road, Mumbai, India) was freshly prepared by dissolving 10 mg in 3 mL of methanol and complete to 100 mL with distilled water. Working solution of the reagent (1 μg mL^−1^) was prepared by further dilution with distilled water. 2-Mercaptoethanol (Alpha Chemika, Mumbai, India) was freshly prepared as 0.5% (v/v) in water. Sodium hydroxide (El-Nasr Chemical Co., Cairo, Egypt) (0.1 M) was prepared by dissolving 400 mg in 100 mL of distilled water. Methanol was obtained from Merck, Darmstadt, Germany. All the reagents used were of analytical grade and the solutions were prepared with double distilled water.

### 2.3 Pharmaceutical formulations

The following available commercial preparation was analyzed; Gaptin® capsules (Delta Pharma S.A.E, 10^th^ of Ramadan City, El Sharkeya, Egypt) labeled to contain 100 mg gabapentin per capsule.

### 2.4 Preparation of standard solution

A stock solution of gabapentin was prepared by dissolving 10.0 mg of the investigate drug in 100 mL methanol. This solution was further diluted with the same solvent as appropriate to obtain the required working concentrations. The standard solutions were stable for at least 7 days when kept in the refrigerator.

### 2.5 General analytical procedure

Aliquots of gabapentin standard solutions covering the working concentration ranges (25–125 ng mL^−1^ as final concentrations) were quantitatively transferred into a series of 10 mL volumetric flasks. To each flask 0.6 mL of 2-mercaptoethanol solution (0.5% v/v) was added, followed by 0.8 mL of 0.1 M sodium hydroxide and mixed well. The solutions were allowed to stand for 10 min. Then, 1.7 mL of *o*-phthalaldehyde was added. The reaction mixture was allowed to stand for 25 min. The volume was completed to the mark with methanol and the fluorescence intensity of the reaction product was measured at 431 nm after excitation at 335 nm. The relative fluorescence intensity was plotted against the final concentration of the drug (ng mL^−1^) to get the calibration graph.

### 2.6 Analysis of pharmaceutical formulations

Twenty capsules containing the investigated drug were accurately weighed and mixed thoroughly. An amount of the powder equivalent to 10 mg of the drug was dissolved in 100 mL methanol. The solution was further diluted with the same solvent and a portion of the resulting solution was subjected for drug analysis using the previously described general analytical procedure. The nominal content of the capsules were calculated using the corresponding regression equation.

## 3. Result and discussion

**Fig. 2:**
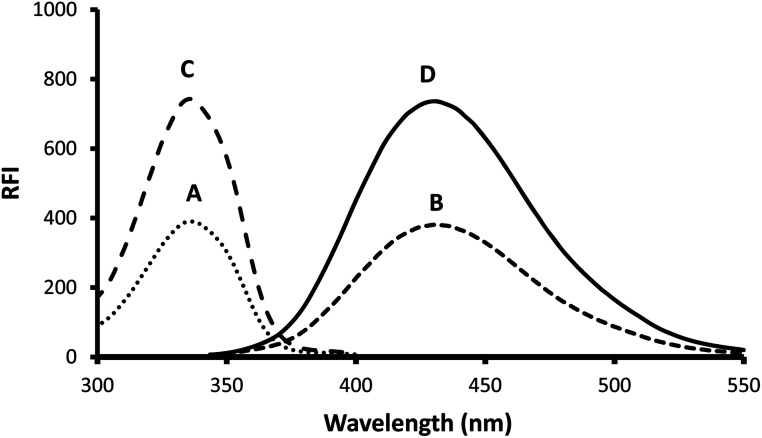
The fluorescence spectra of the reaction product by using 75 ng mL^−1^ gabapentin. Where, A (round dot line): excitation of the blank, B (square dot line): emission of the blank, C (dash line): excitation of the reaction product, and D (solid line): emission of the reaction product.

*o*-Phthalaldehyde in combination with a thiol compound, such as 2-mercaptoethanol, is widely utilized as a fluorescence derivatizing agent for amino compounds. This approach has been applied for the determination of several pharmaceutical compounds.^15–17^ A condensation reaction occurs between the aldehydic groups of *o*-phthalaldehyde and primary amine group of gabapentin lead to formation of a high fluorescent reaction product. Addition of 2-mercaptoethanol to the solution is essential to stabilize the formed reaction product. A proposal for the reaction pathway is presented in Fig. 1. The formed product exhibited high fluorescence at 431 nm after excitation at 335 nm. The fluorescence spectra of the reaction product obtained by using 75 ng mL^−1^ of gabapentin against the blank treated in the same manner are shown in Fig. 2.

The developed spectrofluorimetric method has several advantages over the previous reported methods. The current spectrofluorimetric method is characterized by high sensitivity (in ng mL^−1^) compared with Hassan *et al.*^5^ and Prasad *et al.*^7^ methods. Also, the method is cheap as it does not depend on using expensive reagent compared with Belal *et al.* method.^6^ In addition, the proposed spectrofluorimetric method is highly simple as there is no need for heating, using buffer or extraction with ethyl acetate as in Ulu *et al.* method.^8^ As a result, the current spectrofluorimetric method is a good alternative to the previous reported methods and could be ideally suited for quality control laboratory.

### 3.1 Optimization of the reaction condition

The fluorescence properties of the reaction product, as well as the different experimental parameters affecting the development and stability of the reaction product were carefully studied and optimized. Each factor was changed individually while others were kept constant. The examined factors included; volumes of 2-mercaptoethanol, sodium hydroxide and *o*-phthalaldehyde, reaction time and diluting solvents.

#### 3.1.1 Effect of volume of 2-mercaptoethanol

**Fig. 3:**
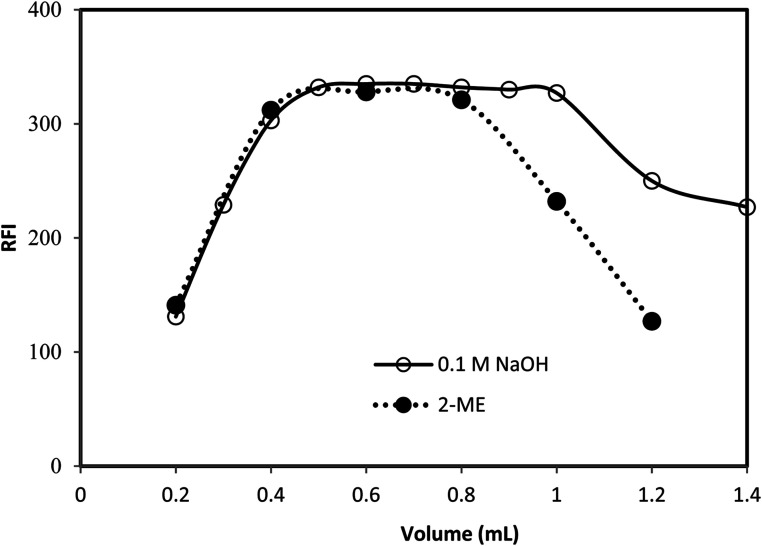
Effect of the volume of 0.5% v/v 2-mercaptoethanol (using 0.8 mL of 0.1 M NaOH) and effect of volume of 0.1 M NaOH (using 0.6 mL of 0.5% v/v 2-mercaptoethanol) on the relative fluorescence intensity of the reaction product of 75 ng mL^−1^gabapentin.

The addition of 2-mercaptoethanol is necessary to stabilize the reaction product of the studied drug with *o*-phthalaldehyde. Different volumes of 0.5% 2-mercaptoethanol solution (0.2–1.2 mL) were used in performing the general analytical procedure. It was found that, increasing the volume of the reagent produces a proportional increase in the fluorescence intensity of the reaction product up to 0.4 mL. The fluorescence intensity remains unchanged up to 0.8 mL (Fig. 3). Therefore, 0.6 mL of 0.5% of 2-mercaptoethanol solution was chosen as the optimal volume of the reagent.

#### 3.1.2 Effect of sodium hydroxide volume

The fluorescence intensity of the reaction products of the investigated drug was examined using different volumes (0.2–1.5 mL) of 0.1 M sodium hydroxide solution. Increasing the reagent volume resulted in increasing the fluorescence intensity. Maximum values were obtained at 0.5 mL of sodium hydroxide and remain constant up to 1.0 mL. However, higher volumes of the reagent gradually decreased the fluorescence intensity of the reaction products. Therefore, 0.8 mL of sodium hydroxide was selected as optimum volume throughout the study (Fig. 3).

#### 3.1.3 Effect of volume of *o*-phthalaldehyde

**Fig. 4:**
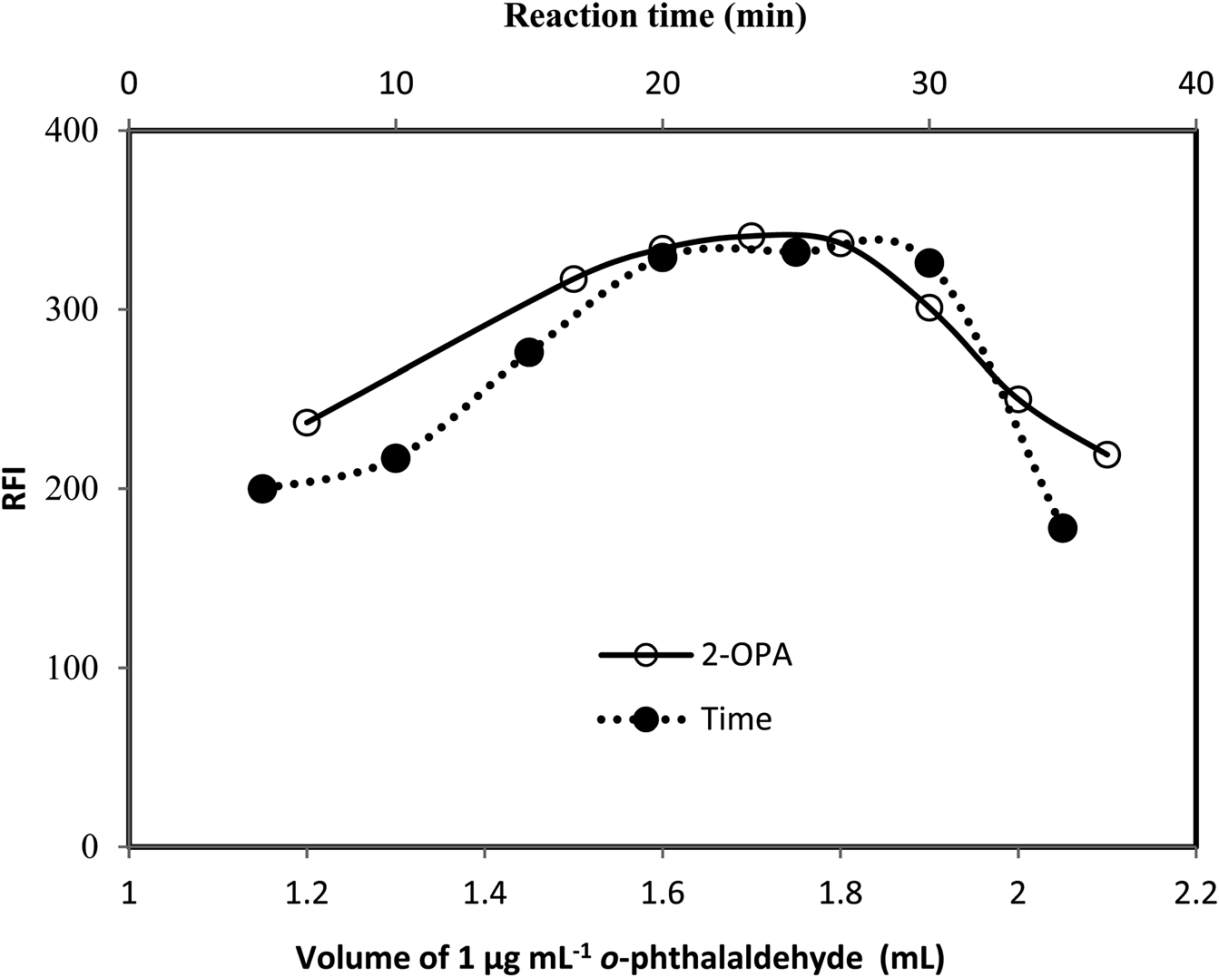
Effect of volume of 1 μg mL^−1^*o*-phthalaldehyde (-○-) at 25 min and effect of reaction time (-●-) using 1.7 mL of 1 μg mL^−1^*o*-phthalaldehyde on the relative fluorescence intensity of the reaction product of 75 ng mL^−1^gabapentin.

The influence of changing *o*-phthalaldehyde volume was studied using different volumes (0.5–2.5 mL) of the reagent (Fig. 4). It was observed that, increasing the volume of the reagent produces a proportional increase in the fluorescence intensity of the reaction product up to 1.6 mL. The fluorescence intensities remained unchanged until l.8 mL after which, a gradual decrease in the fluorescence intensity were observed. Consequently, 1.7 mL of *o*-phthalaldehyde solution was chosen as the optimal.

#### 3.1.4 Effect of reaction time

The reaction was allowed to proceed at different time intervals were tested (Fig. 4) and it was found that maximum relative fluorescence intensity was obtained after 20 min and remains stable up to 30 min. Hence, allowing the reaction mixture to stand for 25 min was adequate for maximum fluorescence intensity.

As, the fluorescence intensity is directly proportional to the fluorescent product concentration. By increasing the reaction time, volume of 2-mercaptoethanol, sodium hydroxide or *o*-phthalaldehyde, the concentration of the fluorescent product (fluorophore) increase and subsequently the fluorescent intensity increased up to saturation. Further increasing in the fluorophore concentration, the fluorophore absorbs the exciting and possibly emitted light (the inner filter effect), reducing the amount of light detected and hence reduces the fluorescence intensity.

#### 3.1.5 Effect of diluting solvent

**Fig. 5:**
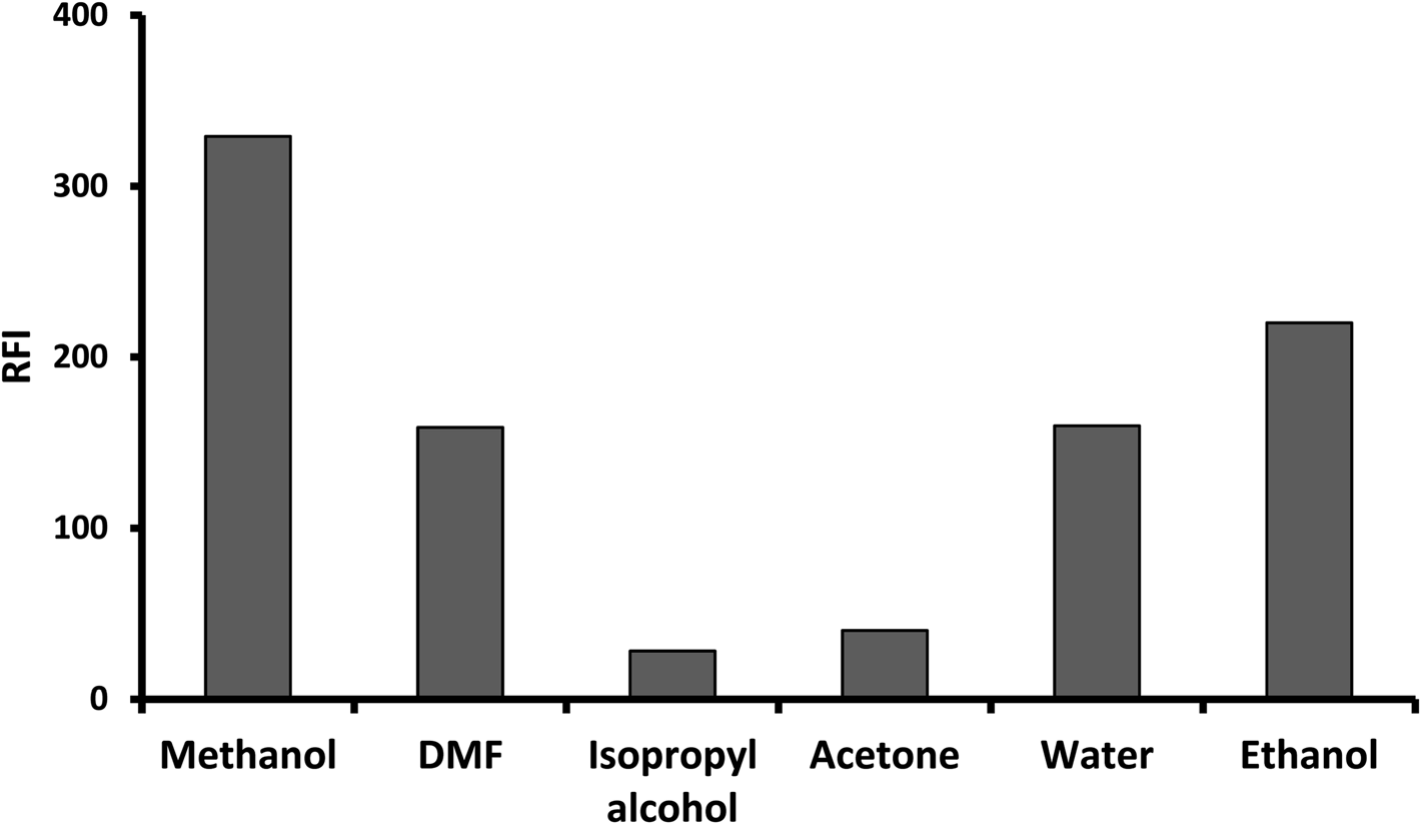
Effect of different solvent on the relative fluorescence intensity of the reaction product of 75 ng mL^−1^ gabapentin.

The formed fluorophore was diluted with different solvents. The examined solvents included; water, methanol, ethanol, isopropyl alcohol, dimethyl formamide and acetone (Fig. 5). Of all the studied solvents, the highest fluorescence intensity was obtained upon using methanol. The variable effect of different types of solvents on the fluorescence intensity of the formed product could be attributed to that the changes in the solvent polarity and the surrounding environment have a great effect on the formed fluorophore.

### 3.2 Validation of the proposed method

According to ICH Q2 recommendations,^18^ the analytical validity of the method was tested regarding; linearity, sensitivity, accuracy, and precision.

Linearity and range: the calibration curve for studied drug was constructed by plotting the relative fluorescence intensities (RFI) against the final investigated drug concentrations (ng mL^−1^). Linear regression analysis of the data was performed and the calculated analytical parameters including slope, intercept, standard deviation of slope and intercept and correlation coefficient are summarized in Table 1. From the calibration curves, we could observe that the RFI values and the drug concentrations was linear dependent within the range of 25–125 ng mL^−1^ of gabapentin. The excellent linearity of the proposed method was indicated by the high correlation coefficients. In addition, the small values of standard deviation of the intercept (*S*_a_), and standard deviation of the slope (*S*_b_) indicate low scattering of the points around the calibration curves.

**Table 1:** Analytical parameters for determination of the studied drug using the proposed method

Parameters	Value
Linear range (ng mL^−1^)	25–125
Intercept (*a*)	69.8
Standard deviation of the slope (*S*_b_)	0.046
Slope (*b*)	3.416
Standard deviation of the intercept (*S*_a_)	3.849
Correlation coefficient (*r*)	0.9997
Limit of detection (LOD, ng mL^−1^)	3.4
Limit of quantitation (LOQ, ng mL^−1^)	11.2

Accuracy: in general, the accuracy could be defined as the closeness or agreement of the obtained analytical value to the true value and measured by calculating the percentage (%) recoveries. For the proposed method, the accuracy was checked by preparation of three standard solutions containing different concentrations of the studied drug within the specified linear range. The concentration of each standard solution was measured by our proposed method in triplicate manner. As shown in Table 2, the obtained % recoveries are close to 100% which indicates the good accuracy of the proposed method.

**Table 2:** Evaluation of accuracy for the proposed analytical method

No.	Taken (ng mL^−1^)	Found^a^ (ng mL^−1^)	% recovery
1	25	25.43	101.72
2	75	74.41	99.21
3	125	123.98	99.18
Mean			100.37
SD^b^			1.458
% RSD^b^			1.457

a The value is the average of three determinations.

b SD is the standard deviation and % RSD is the relative standard deviation percentage.

Precision: the precision is closeness or agreement of obtained analytical values to each other and is measured by calculating the relative standard deviation (RSD). The intra- and inter-day precisions were evaluated by applying the general analytical procedure for the analysis of standard drug solutions having three different concentrations within the specified linear range. The analysis was repeated three times within the same day for the intra-day precision and at three successive days for inter-day precision. The results were expressed in the form of % recovery and % relative standard deviation (Table 3). The calculated relative standard deviations were found to be very small and below 2%, indicating good repeatability and reliability of the proposed method.

**Table 3:** Evaluation for the precision of proposed method for determination of gabapentin

Conc. level (ng mL^−1^)	Intra-day precision		Inter-day precision	
	% recovery ± SD^a^	% RSD^a^	% recovery ± SD^a^	% RSD^a^
25	98.59 ± 1.17	1.19	100.94 ± 1.17	1.15
75	99.74 ± 1.62	1.63	98.96 ± 1.62	1.64
125	100.28 ± 1.17	1.17	100.85 ± 0.62	0.61

a SD – standard deviation, % RSD – relative standard deviation percentage.

Sensitivity: the sensitivity of the proposed method was evaluated by calculating the limit of detection (LOD) and limit of quantitation (LOQ). Based on the standard deviation of the intercept (*σ*) and the slope of calibration curve (*S*), the Limit of detection (LOD) and limit of quantitation (LOQ) were calculated using the following formula (LOD = 3*σ*/*S* and LOQ = 10*σ*/*S*). The obtained detection and quantitation limits were 3.4 and 11.2 ng mL^−1^, respectively (Table 1). These values indicate the high sensitivity of the proposed method.

Robustness of the proposed method was evaluated by introducing minor changes in the experimental parameters such as volumes of *o*-phthalaldehyde, 2-mercaptoethanol, and sodium hydroxide and the reaction time (Table 5). These minor changes have no effect on the method performance as the obtained fluorescence intensities were nearly the same. As a result, the proposed method could be considered as robust.

**Table 4:** Analysis of gabapentin commercial capsule using the proposed spectrofluorimetric and reported methods

Commercial capsules	% recovery ± SD (*n* = 5)		*t* value^a^	*F* value^a^
	Proposed method	Reported method		
Gaptin® capsules	99.93 ± 1.71	99.79 ± 0.92	0.171	3.448

a Tabulated values at 95% confidence limit; *t* = 2.306 and *F* = 6.338.

**Table 5:** Robustness for the proposed analytical method

Parameters	Change	% recovery ± SD
*o*-Phthalaldehyde volume	1.6 mL	99.11 ± 1.27
	1.8 mL	101.25 ± 1.35
2-Mercaptoethanol volume	0.4 mL	99.32 ± 1.16
	0.8 mL	101.45 ± 1.23
Sodium hydroxide volume	0.5 mL	99.83 ± 1.18
	1.0 mL	101.23 ± 1.26
Reaction time	20 min	99.93 ± 0.76
	30 min	101.33 ± 0.87

### 3.3 Application to pharmaceutical capsules

The general analytical procedure was applied for the quantitative analysis of commercial capsules containing gabapentin. The obtained results were statistically compared with those of the reported method^3^ using *t*- and *F*- tests. At 95% confidence level, no significant difference was observed between the calculated and theoretical values of both parameters indicating the acceptable level of precision and accuracy of the proposed method (Table 4). It is clear from the analysis of the dosage forms that the presence of excipients has no significant interference in the results of the analysis. This indicates that the proposed method could be used for determination of the investigated drug in their commercial capsules with a high degree of accuracy, precession and selectivity.

## 4. Conclusion

A highly sensitive procedure for the selective spectrofluorimetric determination of gabapentin through derivatization with *o*-phthalaldehyde was developed and validated. The proposed method was successfully applied for determination of investigated drug in their commercial pharmaceutical capsules with good accuracy and precision without any interference from common excipients. The involved methodology does not require elaborate treatment for the sample or tedious extraction steps. The high sensitivity together with the recognized advantage of the relatively low cost of spectrofluorometric instrumentation make the application of the proposed method is feasible in routine analysis. Therefore, the proposed method is ideally suited for determination of gabapentin in quality control laboratories.

## Conflicts of interest

There are no conflicts to declare.

## Supplementary Material
